# Plasma-Assisted Synthesis of Platinum Nitride Nanoparticles under HPHT: Realized by Carbon-Encapsulated Ultrafine Pt Nanoparticles

**DOI:** 10.3390/nano10091780

**Published:** 2020-09-09

**Authors:** Jian Zhang, Lin Lin, Hang Cui

**Affiliations:** 1 College of Science, Beihua University, Jilin 132013, China; zhangjian_beihua@126.com; 2 Jilin Provincial Key Laboratory of Wooden Materials Science and Engineering, Beihua University, Jilin 132013, China; 3 State Key Laboratory of Superhard Materials, College of Physics, Jilin University, Changchun 130012, China; cuihang@jlu.edu.cn

**Keywords:** plasma-assisted direct-current arc discharge, noble metal nitrides, high pressure and temperature, laser-heating, diamond anvil cell

## Abstract

Noble metal nitrides (NMNs) have important theoretical significance and potential application prospects due to their high bulk modulus and remarkable electrical properties. However, NMNs can only be synthesized under extreme conditions of ultrahigh pressure and temperature, and nanoscaled NMNs have not been reported. In this work, as typical NMNs, PtN_x_ nanoparticles were synthesized at 5 GPa and 750 K by the method of plasma-assisted laser-heating diamond anvil cell. The significantly reduced synthesis condition benefited from the ingenious design of the precursor and the remarkable chemical activity of the ultrafine Pt nanoparticles. This study, combining nanomaterials with high-pressure and -temperature (HPHT) techniques, provides a novel process for the preparation of NMN nanomaterials, and a new direction for the synthesis of superhard materials.

## 1. Introduction

The design of new materials with hardness comparable to that of diamonds are of great technological importance and pose a considerable experimental challenge [1,2,3,4,5,6]. Platinum (Pt) and nitrogen (N_2_) were regarded as lazy materials due to their excellent chemical stability, which makes it difficult for them to react with other elements except under specific conditions [7]. The discovery of platinum nitride (PtN_x_), a potential superhard material and the first binary noble-group nitride, opens the door to new nitrogen chemistry under extreme conditions. In 2004, PtN_x_ was synthesized by compressing Pt and N_2_ at pressures of 50 GPa and temperatures above 2000 K in a diamond anvil cell (DAC) [8]. It was assigned to a ZnS-type structure with a bulk modulus of 372 GPa, which is much higher than that of pure Pt metal (270 GPa). This work emphasized the intriguing possibilities regarding the synthesis of other nitrides with notable or distinctive properties under extreme conditions. Many theoretical investigations were performed to explore the structure of Pt–N systems, and a family of noble metal nitrides were synthesized. In 2006, Crowhurst et al. synthesized IrN_2_ at 48 GPa and 1600 K by laser-heating DAC techniques [9]. Young et al. prepared IrN_2_ (64 GPa and 1800 K) and OsN_2_ (43 GPa and 1800 K) using a similar high-pressure and high-temperature (HPHT) method, and their bulk modulus (IrN_2_: 428 GPa, OsN_2_: 358 GPa) was measured [10]. After that, PdN_2_ was also successfully synthesized at 58 GPa and 1000 K [11]. Meanwhile, some oddities were encountered in intense theoretical efforts [12,13,14,15,16,17]. For example, the molar volume of PtN_x_ is larger than that of the starting materials at pressures above 12 GPa, which is in contradiction with Le Chatelier’s law. X-ray diffraction was unable to provide insight into the internal position of the N atoms or into the stoichiometry of PtN due to the large Pt/N atomic mass ratio. The NaCl-type structure became more dominant than the ZnS-type structure in the pressure range of 4–18 GPa. Nonetheless, a well-recognized fact was that binary noble metal nitrides could be synthesized in extreme conditions; the long-held belief that noble metals do not develop nitrides was broken. However, such extreme conditions severely limit laboratory synthesis and further applications. A method that can significantly reduce the synthesis conditions is desirable.

Material systems of reduced size or dimensionality may, and often do, exhibit properties different from those found in the bulk [18]. Research on ultrafine particles (UFPs) has been fairly active for the last few decades due to the high potential for future technical applications [19,20,21]. However, UFPs could be oxidized or even self-ignited when exposed to air due to extreme chemical activity [22]. Carbon shows a unique ability to form a wide variety of hollow-cage structures. These “nanocapsules” are airtight and protect the encapsulated materials from hydrolysis and oxidation [23]. Since the pioneering work of Ruoff et al., the traditional plasma-assisted arc discharge method has been widely used in the preparation of various carbon-encapsulated nanocrystals [24]. However, not all materials could be encapsulated into carbon cages, and most of them are in the form of their carbides [25]. Elements were divided into three classes by Seraphin, either by considering their composite or bonding mode: (I) encapsulated in the form of carbides such as B, V, Cr, Mn, Y, Zr, Nb, and Mo; (II) encapsulated in the form of elements such as Fe, Co, and Ni; or (III) unable to be encapsulated such as Pd, Ag, and Pt [26].

In this work, we offer a novel two-step method to synthesize carbon-encapsulated Pt UFPs, and then to synthesize PtN_x_ nanomaterials under relatively mild synthesis conditions. The significantly reduced synthesis condition benefited from the ingenious design of the precursor and the remarkable chemical activity of Pt UFPs. This work strongly suggests that it could be possible to synthesize other noble-group nitrides by combining the arc discharge method with HPHT. We expect that this study will not only help to further investigate the undisclosed intrinsic physical properties of PtN_x_, but might also shed some light onto the synthesis of other potential superhard materials.

## 2. Materials and Methods 

### 2.1. Materials

Platinum foils (Pt, 99.999%) were purchased from Sigma-Aldrich (St. Louis, MO, USA). A tungsten rod (W, 99.995%), graphite rod (C, 99.995%), and graphite crucible (C, 99.995%) were purchased from Alfa-Aesar Company (Beijing, China). Helium (He, purity: 99.999%), argon (Ar, purity: 99.999%), and nitrogen (N_2_, purity: 99.999%) were purchased from Sinopharm Company (Shanghai, China). All chemicals were used as received.

### 2.2. Synthesis

#### 2.2.1. Preparation of Platinum Nanoparticles

**Figure 1:**
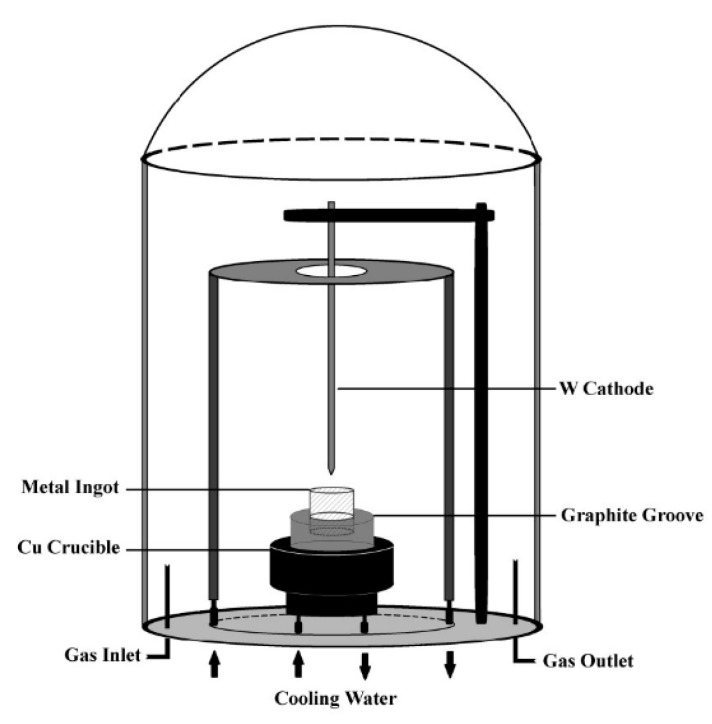
A schematic diagram of the experimental set-up.

The plasma-assisted arc discharge process was performed in a modified experiment apparatus that consisted of a stainless-steel chamber with two electrodes facing each other, lined up in a vertical configuration. A schematic diagram of the experiment setup is shown in Figure 1 [27]. A tungsten rod (purity: 99.995%, 5 mm diameter and 30 cm length) was at the top as the cathode. Platinum foils (purity: 99.999%, 10 mm thickness and 30×30 mm size) were nipped by platinum arms that were fixed inside a water-cooled copper crucible as the anode. After the chamber was evacuated, helium gas (He, purity: 99.999%) was introduced as a working gas to reach the desired pressure. During arc discharge (18 V voltage, 120 A current, and 40 kPa helium pressure), platinum foils were evaporated, and the gap between the electrodes was kept constant (1 mm) by manually advancing the consumed anode. This process was maintained for 2 min; after that, the evaporated materials were quenched by helium for 5 h and deposited on the wall of the chamber.

#### 2.2.2. Preparation of Carbon-Encapsulated Ultrafine Platinum Nanoparticles

The as-synthesized platinum nanoparticle powders were pressed into a metal block and placed inside a graphite crucible with an inner diameter of about 10 mm as the anode. The graphite rod (purity of 99.995%, 5 mm diameter and 30 cm length), instead of the tungsten rod, was located at the top as the cathode. After that, the chamber was first evacuated to less than 1 Pa and then filled with argon gas. This cycle was repeated several times to completely remove the residual air, and the working gas (Ar, purity: 99.999%, 10 kPa) was then introduced into the chamber. During arc discharge, the current was maintained at 80 A, and the voltage was 20 V. After discharging for 10 min, the products were passivated for 6 h in the Ar atmosphere. The fluffy dark deposits were collected from the wall of the chamber.

#### 2.2.3. High-Pressure Experiment and Raman Measurement

A Mao–Bell-type diamond anvil cell (DAC) (Almax easyLab company, Ashford, UK) with a culet diameter of 400 μm was used for a laser-heating high-pressure experiment. A preindented rhenium slice (Alfa-Aesar Company, Beijing, China) with thickness of 40 μm served as a gasket material. A 200 μm diameter hole was drilled into the center of the gasket to form a sample chamber. The second discharge synthesized sample powder (carbon-encapsulated ultrafine platinum nanoparticles) was loaded into the chamber with a small ruby ball (Almax easyLab company, Ashford, UK). The pressure was determined from the frequency shift of the ruby R1 fluorescence line. The pressure was measured before and after heating and did not appreciably change. As a quasi-hydrostatic pressure medium, nitrogen (N_2_, purity: 99.999%) was transformed into liquid and loaded into the DAC under low-temperature conditions. The DAC was warmed to room temperature when adequate pressure was applied. High-pressure Raman measurements were performed at room temperature using a solid-state diode-pumped Nd:vanadate laser with 532 nm wavelength as the excitation source. An Acton SpectraPro 500i spectrometer with 1800 gr/mm holographic grating and a liquid-nitrogen-cooled charge-coupled device (CCD) detector with format of 1340 × 100 were used.

#### 2.2.4. Laser-Heating Synthesis of PtN_x_

A double-sided laser-heating system was used for a laser-heating high-pressure experiment. The Nd:YLF laser (55 W, λ = 1053 nm) could provide a beam with a low divergence and high stability. The beam was split into two parts by a 50/50 high-energy laser prism, and then focused onto the sample from two sides by achromatic objective lenses through diamonds. The sample image and its thermal radiation were collected and focused onto a CCD detector with an achromatic lens. A 50/50 beam splitter was used to split the beam into two branches. The transmitted branch was connected to a monitor, and the reflected branch was used for temperature measurements. The temperature was measured with a thermoelectrically cooled CCD detector equipped with an Acton SpectraPro 300i spectrograph.

### 2.3. Characterization

The crystal structure of the samples was collected on a D/max γA diffractometer using a Cu–Kα target (λ = 0.154178 nm) (XRD, MiniFlex-600, Rigaku Co., Tokyo, Japan). The morphology of the samples was observed by field-emission scanning electron microscopy (SEM, Magellan-400, FEI Co., Hillsboro, OR, USA). The single-crystalline feature and elemental distribution were verified by the transmission electron microscope using an accelerating voltage of 200 kV (TEM, HRTEM(High Resolution Transmission Electron Microscopy), and Mapping, JEM-2200FS, JEOL Co., Tokyo, Japan). The characteristic modes of the sample were measured by a Raman system that consisted of the solid-state diode-pumped Nd:vanadate laser (Coherent Co., Santa Clara, CA, USA) and the liquid-nitrogen-cooled CCD detector (Princeton Co., Trenton, NJ, USA).

## 3. Results and Discussion

**Figure 2:**
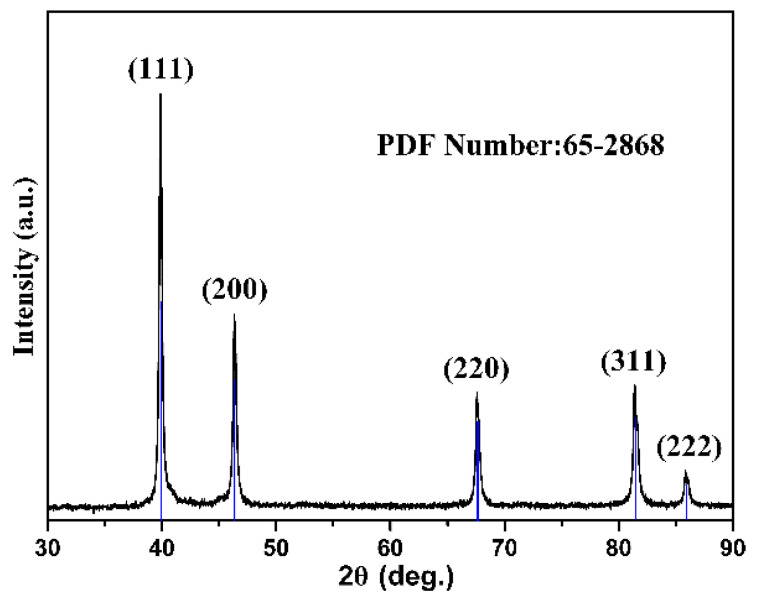
XRD pattern of Pt nanoparticles.

The XRD pattern recorded for the as-synthesized sample is depicted in Figure 2. All diffraction peaks could be indexed to a cubic structure with the space group Fm-3m. The lattice parameter of a = 3.924 Å was in good agreement with the standard card (JCPDS no. 65-2868). Diffraction peaks corresponding to any kind of impurities were not observed in the XRD pattern. The average particle size was evaluated as 25 nm using Scherrer’s formula. The peaks were sharp instead of wide, indicating the dominance of the bulk materials.

**Figure 3:**
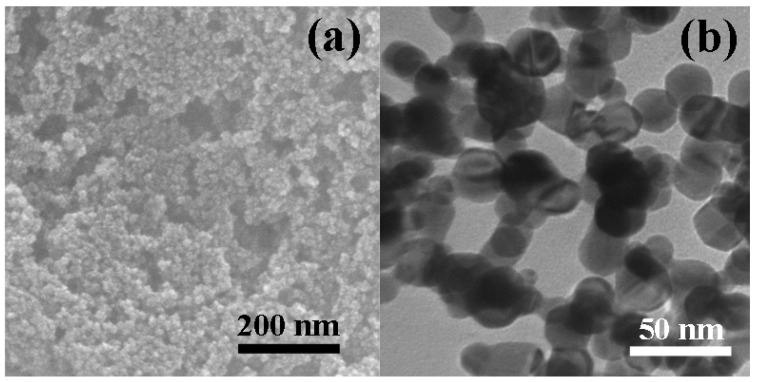
(**a**) SEM and (**b**) TEM of Pt nanoparticles.

A typical SEM image of the as-synthesized sample is shown in Figure 3a, which mostly exhibited a uniform spherical morphology. From the TEM image in Figure 3b, it is apparent that the nanoparticles had a smooth surface and a diameter of 20–30 nm, which is consistent with the previous XRD data.

**Figure 4:**
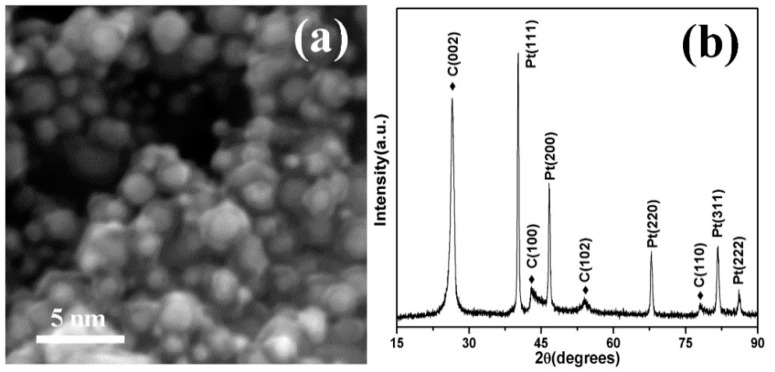
(**a**) SEM and (**b**) XRD of carbon-encapsulated ultrafine Pt nanoparticles.

Further reducing the size of Pt nanoparticles is the critical factor to synthesize platinum nitrides under relatively mild conditions. To reserve the chemical activity of ultrafine nanoparticles (UFPs), carbon-encapsulated Pt UFPs were synthesized with a method using the secondary discharge of preprepared Pt nanoparticles. Figure 4a is the SEM image of the carbon-encapsulated Pt UFPs; the outer diameter of the encapsulated nanoparticle was about 5 nm, and the size of the inner Pt UFPs was less than 2 nm. The XRD pattern in Figure 4b shows that there were no other diffraction peaks except for C and Pt, which means that the carbon was wrapped around the Pt UFPs rather than forming platinum carbides. The widened peaks also indicate the particle size is small.

**Figure 5:**
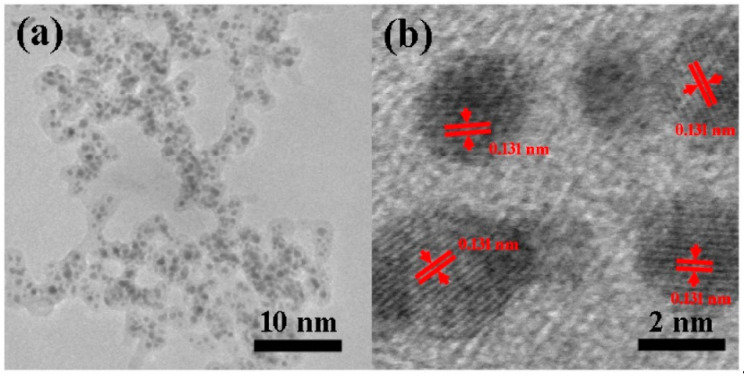
(**a**) TEM and (**b**) HRTEM of carbon-encapsulated ultrafine Pt nanoparticles.

Encapsulation could be observed more directly by TEM. As shown in Figure 5a, Pt UFPs are separated from each other by the carbon shell. To further elucidate the structure of the central nanoparticles, a corresponding HRTEM were performed. As shown in Figure 5b, Pt UFPs with a diameter of 2 nm were confirmed as single-crystal; the distance between adjacent lattice planes was about 0.131 nm, which agreed well with the d-spacing of cubic Pt. It means that the central nanoparticle remained as a metal rather than carbide after the secondary discharge process, which was consistent with the SEM results. Meanwhile, the potential chemical activity of Pt UFPs was confirmed.

**Figure 6:**
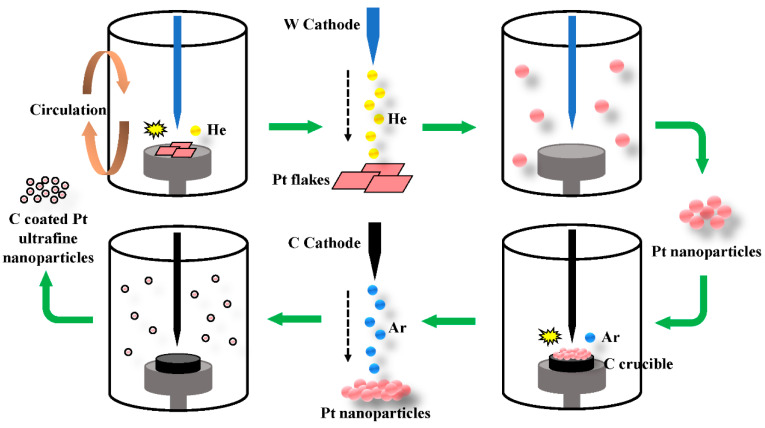
Growth mechanism of carbon-encapsulated ultrafine Pt nanoparticles.

In terms of the sample characterizations and arc discharge features, a possible growth mechanism of the carbon-encapsulated Pt UFPs is proposed as illustrated in Figure 6. When the arc discharge was ignited, the Pt foils were struck by high-energy plasma helium (He) and drastically evaporated in the central high-temperature zone. With circulation, Pt vapors were transported by the He carrier gas to the water-cooled collecting wall and formed 25 nm Pt nanoparticles. To further reduce the size of Pt nanoparticles and prepare the carbon-encapsulated Pt UFPs, a second discharge was performed. In this process, the tungsten rod cathode was replaced by the carbon rod, and the as-synthesized Pt nanoparticle powder was slightly pressed into an ingot as a precursor that was placed inside a graphite crucible as the anode. The carbon rod cathode and graphite crucible anode played a role in providing a carbon source. To raise the reaction temperature, the working gas was replaced by argon (Ar). When discharged again, the Pt nanoparticles were hit by Ar plasma, and formed the 5 nm carbon-encapsulated Pt UFPs on the surface of the water-cooled collecting wall. The crucial preparation factor of carbon-encapsulated Pt UFPs was the ingenious design of the precursor. In the traditional arc discharge method, encapsulated objects are usually bulk materials that discharge only once. In this work, nano-treated precursors had characteristics of a higher chemical activity, lower melting point, and easier reassembly of UFPs, which allowed for their recombination with carbon into a core–shell structure.

**Figure 7:**
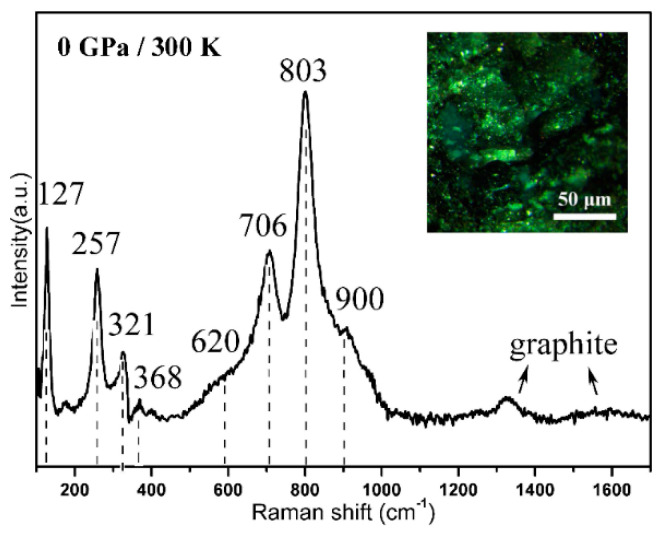
Raman spectra of PtN_x_. Measurements done at ambient condition (inset: quenched sample in opened diamond anvil cell (DAC) chamber).

Raman spectroscopy permits the identification of unknown materials from characteristic modes. For noble-group nitrides, Raman is a common method of determining whether the bonding between metal and nitrogen was formed because of pure metal that had no characteristic modes. In this work, liquid nitrogen (N_2_) was the pressure medium and nitrogen source, and the Pt UPF powder was used as a laser absorber in the DAC experiment. As shown in Figure 7, following the laser-heating experiment at a pressure of 5 GPa and temperature of 750 K, a Raman measurement of the quenched sample was performed. The Raman modes were clear, and at least eight modes (127, 257, 321, 368, 620, 706, 803, and 900 cm^−1^) of PtN_x_ were detected; the remaining modes are expected at low frequency ranges or to have low intensities. The Raman spectra measured in the entire section were identical through point-by-point detection in the opened DAC chamber, indicating that the products had a well-crystallized and highly ordered structure. For bulk PtN_x_, dominant Raman modes are located at 740 and 870 cm^−1^ [8,9]. In this work, the major peaks of nano-PtN_x_ were shifted to 706 and 803 cm^−1^, respectively, and show an obvious widening signal. In general, the widening and blue shift of the Raman modes in nanomaterials are the most important characteristics compared with their corresponding bulk materials which is attributed to the size confinement effect [28]. In addition, the large shift was also related to the high-pressure because of the Raman modes move with the pressure changes. The synthetic pressure of nano-PtN_x_ is only 5 GPa and that of the bulk PtN_x_ reaches more than 50 GPa—the degree of sluggishness phenomenon is different during decompression. The higher the pressure, the more obvious the phenomenon [29,30]. Meanwhile, many new modes were observed below 700 cm^−1^ for the first time. Because there were only three elements (Pt, C, and N) in the DAC sample chamber for laser heating—the modes of N–N at 2322 cm^−1^, C at 1350 and 1580 cm^−1^ (arrows in Figure 7), and the pure Pt metal without Raman modes—these modes were identified as a Pt–N mode [31,32].

**Figure 8:**
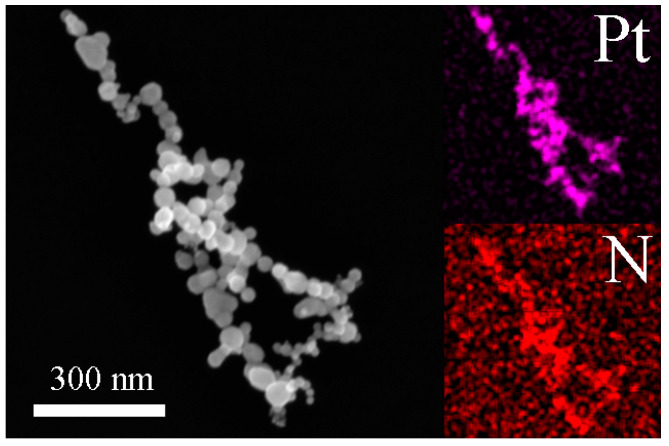
Chemical analysis of quenched sample.

To identify the elemental distribution and Pt/N atom ratio, a mapping analysis measurement was performed. As shown in Figure 8, the quenched sample still spherical nanoparticles with a particle size of 30–80 nm, which was larger than before. Two elements were homogeneously distributed in each particle and exhibited no apparent elemental separation or aggregation. The atomic ratio of Pt/N was close to 1:1 but with some variations, that is, PtN_1–x_ with x < 0.5. This was attributed to the mapping analysis mainly being done from the sample surface, either pure PtN_x_ in most locations or PtN_x_ plus unreacted Pt in unreacted zones.

**Figure 9:**
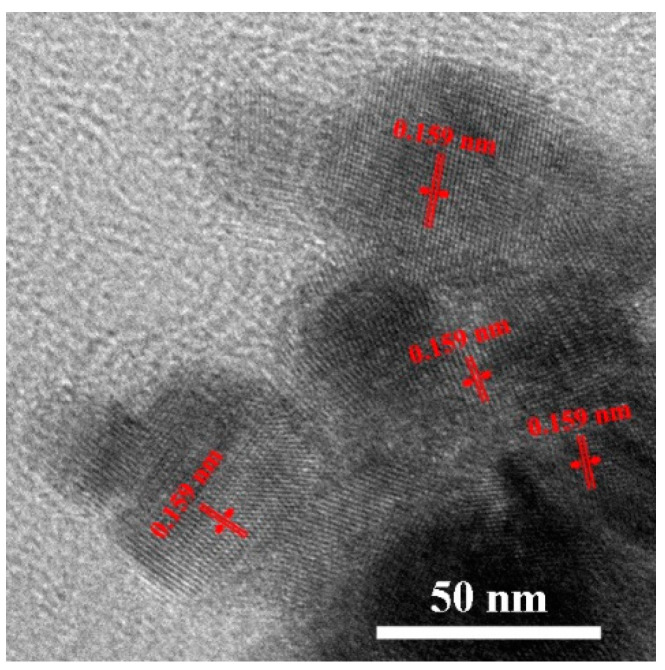
Chemical analysis of quenched sample.

To further elucidate the microstructure of PtN_x_, HRTEM investigations were performed. As shown in Figure 9, PtN_x_ nanoparticles present a series of clear lattices and which were confirmed as single-crystal; the distance between adjacent lattice planes was about 0.159 nm, which is larger than Pt UFPs and agreed well with the d-spacing of Zn-S type PtN_x_. It means that PtN_x_ nanoparticles are still in the cubic crystal system as metal Pt, which is consistent with previous reports [8,9].

**Figure 10:**
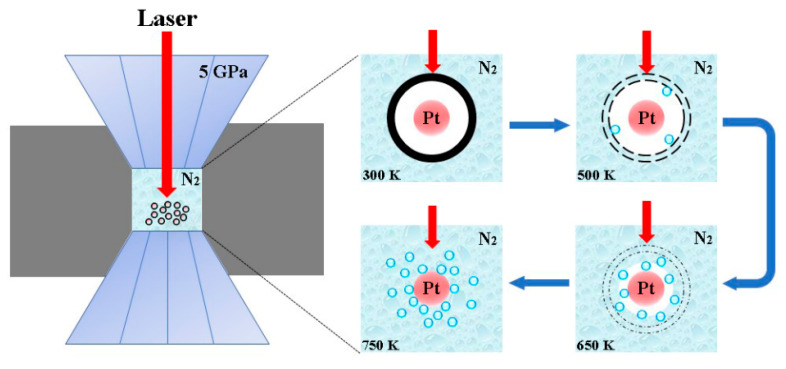
Growth scheme of PtN_x_ nanoparticles.

On the basis of structural characterizations and morphological features, a possible growth mechanism of the PtN_x_ nanoparticles is proposed in Figure 10. When the pressure was elevated up to 5 GPa, the laser focused on the surface of the carbon-encapsulated Pt UPFs. At lower pressure levels, the nitridation reaction was not observed, even at ultrahigh temperatures; at 5 GPa, the reaction proceeded rapidly. With increasing temperature, the intact carbon shell at 300 K started to break down. The surrounding liquid nitrogen flowed into the nanocapsule when cracks appeared in the carbon shell. At 650 K, most of the carbon shell had been destroyed by laser heating, and large amounts of liquid nitrogen flooded into the nanocapsule and approached the Pt UPFs. With the temperature up to 750 K, carbon shells were entirely decomposed, and the Pt UPFs were completely exposed to the liquid nitrogen. Pt UPFs met N molecules to form PtN_x_ nanoparticles under HPHT conditions through the following reaction: Pt(s) + N(l) → PtN_x_(s). When the interior Pt UPFs had been completely consumed, the PtN_x_ nanoparticles were formed.

## 4. Conclusions

We synthesized PtN_x_ nanoparticles at 5 GPa and 750 K via plasma-assisted laser-heating high-pressure and -temperature diamond anvil cell techniques. The crystal structure, morphology, elemental distribution, Raman scattering, and growth mechanism of PtN_x_ nanoparticles were investigated. Compared with traditional methods of binary noble-group nitrides, the synthesis conditions were significantly reduced. This was attributed to the ingeniously designed precursor, and the remarkable chemical activity of ultrafine Pt nanoparticles. By combining the small size effect of the ultrafine nanoparticles with the extreme conditions of the laser-heating DAC technique in this work, we proposed a novel approach for synthesizing nanoscaled noble metal nitrides and a new idea for discovering superhard materials. Our results have important implications for high-pressure experiments and theoretical studies.

## References

[1] Tian Y.J., Xu B., Yu D.L., Ma Y.M., Wang Y.B., Jiang Y.B., Hu W.T., Tang C.C., Gao Y.F., Luo K. (2013). Ultrahard nanotwinned cubic boron nitride. Nature.

[2] Mao H.K., Chen X.J., Ding Y., Li B., Wang L. (2018). Solids, liquids, and gases under high pressure. Rev. Mod. Phys..

[3] Lu C., Li Q., Ma Y.M., Chen C.F. (2017). Extraordinary Indentation Strain Stiffening Produces Superhard Tungsten Nitrides. Phys. Rev. Lett..

[4] Amiri M., Beheshtian J., Shayeganfar F., Faghihnasiri M., Shahsavari R., Ramazani A. (2020). Electro-optical properties of monolayer and bilayer pentagonal BN: First principles study. Nanomaterials.

[5] Zhang R.F., Legut D., Lin Z.J., Zhao Y.S., Mao H.K., Veprek S. (2012). Stability and strength of transition-metal tetraborides and triborides. Phys. Rev. Lett..

[6] Li K.Y., Wang X.T., Zhang F.F., Xue D.F. (2008). Electronegativity identification of novel superhard materials. Phys. Rev. Lett..

[7] Appen J., Lumey M.W., Dronskowski R. (2006). Mysterious platinum nitride. Angew. Chem. Int. Edit..

[8] Gregoryanz E., Sanloup C., Somayazulu M., Badro J., Fiquet G., Mao H.K., Hemley R.J. (2004). Synthesis and characterization of a binary noble metal nitride. Nat. Mater..

[9] Crowhurst J.C., Goncharov A.F., Sadigh B., Evans C.L., Morrall P.G., Ferreira J.L., Nelson A.J. (2006). Synthesis and characterization of the nitrides of platinum and iridium. Science.

[10] Young A.F., Sanloup C., Gregoryanz E., Scandolo S., Hemley R.J., Mao H.K. (2006). Synthesis of novel transition metal nitrides IrN_2_ and OsN_2_. Phys. Rev. Lett..

[11] Crowhurst J.C., Goncharov A.F., Sadigh B., Zaug J.M., Aberg D., Meng Y., Prakapenka V.B. (2008). Synthesis and characterization of nitrides of iridium and palladium. J. Mater. Res..

[12] Sahu B.R., Kleinman L. (2005). PtN: A zinc-blende metallic transition-metal compound. Phys. Rev. B.

[13] Uddin J., Scuseria G.E. (2005). Structures and electronic properties of platinum nitride by density functional theory. Phys. Rev. B.

[14] Kanoun M.B., Goumri-Said S. (2005). Electronic properties of the binary noble metal nitride PtN: First-principles calculations. Phys. Rev. B.

[15] Yu R., Zhang X.F. (2005). Platinum nitride with fluorite structure. Appl. Phys. Lett..

[16] Yu R., Zhan Q., Zhang X.F. (2006). Elastic stability and electronic structure of pyrite type PtN_2_: A hard semiconductor. Appl. Phys. Lett..

[17] Fan C.Z., Sun L.L., Wang Y.X., Wei Z.J., Liu R.P., Zeng S.Y., Wang W.K. (2005). First-principles study on the elastic properties of platinum nitride. Chin. Phys. Lett..

[18] Pascual J.I., Mendez J., Gomez-Herrero J., Baro A.M., Garcia N., Landman U., Luedtke W.D., Bogachek E.N., Cheng H.P. (1995). Properties of metallic nanowires: From conductance quantization to localization. Science.

[19] Richard W.S. (1993). Synthesis and properties of nanophase materials. Mat. Sci. Eng. A.

[20] Gleiter H. (1989). Nanocrystalline materials. Prog. Mater. Sci..

[21] Ryozi U. (1991). Studies of ultrafine particles in Japan: Crystallography. Methods of preparation and technological applications. Prog. Mater. Sci..

[22] Baker C., Hasanain S.K., Shah S.I. (2004). The magnetic behavior of iron oxide passivated iron nanoparticles. J. Appl. Phys..

[23] Saito Y., Yoshikawa T., Okuda M., Fujimoto N., Sumiyama K., Suzuki K., Kasuya A., Nishina Y. (1993). Carbon nanocapsules encaging metals and carbides. J. Phys. Chem. Solids..

[24] Ruoff R.S., Lorents D.C., Chan B., Malhotra R., Subramoney S. (1993). Single crystal metals encapsulated in carbon nanoparticles. Science.

[25] Saito Y. (1995). Nanoparticles and filled nanocapsules. Carbon.

[26] Seraphin S., Zhou D., Jiao J., Wlthers J.C., Loutfy R. (1993). Yttrium carbide in nanotubes. Nature.

[27] Zhang J., Zhu H.Y., Wu X.X., Cui H., Li D.M., Jiang J.R., Gao C.X., Wang Q.S., Cui Q.L. (2015). Plasma-assisted synthesis and pressure-induced structural transition of single-crystalline SnSe nanosheets. Nanoscale.

[28] Gregory J.E., Aimee R., Charles F.W. (1997). Spectroscopic characterization of processing-induced property changes in doped ZnO films. Thin Solid Films.

[29] Wang Z.W., Saxena S.K., Pischedda V., Liermann H.P., Zha C.S. (2001). In Situ X-ray diffraction study of the pressure-induced phase transformation in nanocrystalline CeO_2_. Phys. Rev. B.

[30] Wang Z.W., Saxena S.K., Pischedda V., Liermann H.P., Zha C.S. (2001). X-ray diffraction study on pressure-induced phase transformations in nanocrystalline anatase/rutile (TiO_2_). J. Phys. Condens. Matter.

[31] Pauer F., Kipfstuhl J., Kuhs W.F. (1995). Raman spectroscopic study on the nitrogen/oxygen ratio in natural ice clathrates in the GRIP ice core. Geophys. Res. Lett..

[32] Chung D.D. (2002). Review graphite. J. Mater. Sci..

